# Materials Used in Filling Teeth

**Published:** 1873-04

**Authors:** W. H. Morgan


### "Materials Used in Filling Teeth."

BY W. H. MORGAN, M. D., D. D. S.

*Read before the Tennessee State Dental Association.*

*Mr. President*:—I have recently learned that it is my duty to report to this association upon materials used in filling teeth. Although there is nothing new in the profession on this subject, so far as I am aware—that is, there is no new material, nor any new form of those, in use, save some immaterial modifications—I shall briefly run over the list, and give what I conceive to be the latest and most generally accepted views on the subject, together with my own experience and conclusions:

*Hill's Stopping*. This material, chiefly made of gutta percha and quick lime, has undergone no modification or improvement in its manufacture for years past. The facility with which it can be applied, its tendency to adhere to, and not shrink away from the walls of the cavity when properly applied, together with its cheapness, make it a valuable material for temporary fillings, especially in positions other than the grinding surfaces of teeth. It is especially valuable to protect the pulp during its treatment preparatory to filling, enabling the operator to shut out the fluids of the mouth, or any other irritating substance, from the cavity, during such treatment. It is also used to enclose and shut in any disinfectant used in teeth, the pulps of which have been long dead, and which are consequently offensive to the olfactories. For filling the roots of teeth preparatory to filling the crowns, it is one of the most valuable preparations at the dentist's command; after being warmed properly it may be so manipulated as to answer this purpose; perhaps, more perfectly than any material now used by the profession. I am sorry to say there are hundreds of men in the profession who never use it, indeed who never saw it.

The next article I would call your attention to is oxychloride of zinc and its kindred preparations, including Killint's Cement, etc.

Killint's Cement has proven very unsatisfactory in my hands. I obtained it of Dr. Killint in person, so it was the genuine article; it sets slowly, too slowly, is easily damaged by the fluids of the mouth, and is not sufficiently hard after it sets, being softer than any other preparation of the kind I have used. Next to this comes Roberts' os-artificial, and Smith's os-artificial, of about equal merit, both valuable preparations. Cement Plomb I have not used; it is highly recommended; said by some of our profession, very capable of judging correctly, to be the best of all these preparations. Guillois' Cement is being extensively used, and is of great value; it is more plastic than any of the above, does not set too rapidly, and is harder than any of them, and is of several grades in color.

It is used with success in filling the roots of teeth, and in such as have very weak walls, and is the "*ne plus ultra*" for capping exposed pulps, and partially filling these cavities when the pulp is very nearly approached, and a nonconductor is required. I have never seen but one tooth lost when this filling was used for the latter purpose. Whether its value in capping pulps is due to greater congeniality to that organ or tissue, or to its ready adaptation in form to it, I am not prepared to say; probably to both. I would not know how to practice successfully without it.

Tin foil, perhaps, ranks next; most of you are familiar with its use. There are but two objections to its use, but both of these are well nigh insupurable. First, it blackens, corrodes in the mouth, both when it comes in contact with the fluids of the mouth, and when fluids are infiltrated behind or under it. Second, it is too soft to bear mastication; when used for any length of time will wear out, and needs often to be renewed. Its greatest recommendations are: it is less liable to corrode than any cheap material now in use in pure metalic form, and it is softer. Observation has satisfied me that its softness and lack of maleability often mislead the operator, and he fails in consequence to make a compact filling with it.

Oxidization between the folds of the foil, and decay beneath the plug, consequent upon the permeation of the fluids of the mouth, being often the result, more care and more force is requisite in packing it than are generally used.

Amalgam comes next in order, and is the most valuable of all the cheap materials now in use, except for filling roots, filling over pulps, and for protecting against thermal changes.

With the lights now before me, I am not prepared to recommend any particular make or brand. I know that much of that in use shrinks in hardening, and is useless or nearly so to preserve a tooth. Its excellencies, if you will allow me to use that term, consists in cheapness, ready adaptability to the cavity, and solidity after it hardens; one-half or two-thirds of a tooth can readily be built with it. It requires very little skill to use it, and it were better for all interested, if the major part of our profession, or those calling themselves dentists, would never attempt to fill a tooth with any other material; they have, or may learn to fill a tooth well with this material, they never can with gold, the only other material they pretend to use. But its very cheapness seems to lead to carelessness in its use; every possible care should be taken in the preparation of the cavity for filling; the materials should be carefully ground together, and most thoroughly washed, so as to remove all oxidized matter, the mercury well pressed out, and then packed in the most neat and thorough manner, and when hardened, nicely polished. Perhaps as much as twenty-four hours should elapse before this latter operation is done.

The objections argued against the use of this material for filling teeth are: First, it shrinks, leaving open interstices around the plug, through which foreign matters find their way beneath the filling, and decay is the result; this objection is justly made, although the shrinkage is often so small that the mischief or evil is done slowly. It is claimed that some of the preparations now offered in the market are free from this objection. Doubtful. I have had sad cause to modify my opinions on this subject within the last year. Second, it corrodes and discolors the teeth; this is generally true; always in these cases where it is used in devitalized teeth, and there is some matter passed into the posterior of the filling, either through the foramen at the point of the root or by endosmotic action, and in all cases where there is much oxidization about the margins of the cavity, the secretions and fluids of the mouth will penetrate around the filling, and decay will sooner or later follow. Third, It is claimed that large fillings will produce ptyalism. I do not think this point is well taken; in more than a quarter of a century's constant practice, I have never met with such a case, unless it was a case of local not general ptyalism. I think it probable I have met with a case or two of the latter. Fourth. As it is manifestly improper to use amalgam in the front teeth, it is said it must only be used in such mouths as do not need any filling, requiring gold filling on account of galvanic action. Observation has led me to repudiate this view, as in my extended observation I have never seen any evil effects result from having the two metals in the same mouth, not even in those cases in which they came in actual contact, a large number of which I have seen.

And now, Mr. President, I come to consider the merits of gold, "the king of metals," as a material for filling teeth. There are four prominent objections to it for this purpose: First. The difficulties of manipulating it into a perfect filling, or one so nearly approaching perfection as to preseve the tooth from further decay. According to the best authority I have been able to consult, not more than one in four of the dental practitioners in this country, and a much smaller ratio in Europe, ever attain sufficient skill to use it so that any benefit, even the smallest, is derived by their patients; and the number out of this proportion who use it with any considerable degree of skill, is fearfully small; I am ashamed to suggest their number. It is true, sir, that recent improvements and modifications in preparation of gold for filling teeth have greatly lessened this objection. Second. Its cost: this is a trouble we will perhaps never overcome, the general good of our race would not be greatly promoted thereby. Third. Its color; there is no reasonable expectation that this will ever be corrected, although a few years since some French savant announced that he had succeeded in so enamelling fillings in the mouth as to give them the appearance of the natural teeth. Fourth. Its conducting power; subjecting the pulp of the tooth when the filling is large, and often when small, if very compact, to violent thermal changes, producing pain, irritation, and inflammation of that organ.

Its excellencies are its indestructability, its almost entire freedom from wear by mastication, and its cohesive qualities by which a filling may be made a unit, and by which a large portion, if not the crown of a tooth, may often be permanently restored. The forms in which gold has been used for filling teeth are various: soft foil, or more properly non-adhesive foil, adhesive foil, rolled gold, Eureka filling, the various forms of sponge gold, known as sponge, shred, and plastic gold. Non-adhesive foil has been, and probably now is, the most extensively used of any form of gold; it is not of so universal application to cases, nor can such excellence be obtained in operations as with the other preparations; it has the virtue of being more easily adapted to many of the cases in which it is applicable than any other form of the metal, but so firm a filling can not be made with it as with adhesive gold. Its manufacture has been improved within a few years past, I think, in softness, and certainly in toughness.

I might have added, among the objections to gold, its hardness; I have never seen a sheet of foil yet as soft as I would like it.

Sponge-gold is dependent entirely on its cohesive properties for its value for our purposes, and although it has been, and now is used by some members of our profession exclusively, or nearly so, there have always been serious well-founded objections to it, among which are the difficulty of packing it perfectly against the walls of the cavity, and the impossibity of producing, or procuring a uniform article in quality. The great mass of those who experimented with it, and were disposed to adopt its use in their practice have abandoned it; if not entirely, at least in general. Both shred and plastic gold are but coarser preparations of the same article; the gold being in the same form are liable to the same objections, and have justly shared the same fate as sponge so-called.

Adhesive foil is now more generally used by the more advanced in the profession than any other form of this material. For the sake of convenience, and to save time, I will consider under this head the rolled gold and Eureka filling, as well as adhesive foil.

The rolled gold is used from the ribbon directly from the mill after cleansing and annealing. One manufacturer, Williams, changes the character of the surface from a smooth to one apparently crystaline, by immersing it for a short time in *aqua regia*, thus removing the smoothness left by the rollers. The foil which he makes into blocks is also prepared in this way, and is all very adhesive. The Eureka filling now prepared by Valleau, originally by Geo. S. Pack, is prepared by first drawing the pure gold into wire, then rolling under water, and afterwards annealing. This preparation is proportionately the softest and most adhesive of all the preparations of this metal yet offered to the profession for filling teeth, and for some cases is undoubtedly the best. The first advantage that adhesive has over the non-adhesive, is its universality of application; it can be used in all cases where the non-adhesive can, and in many where it can not, it makes a harder filling; it wears less, may be made to protect frail edges, and restore contour; such is its excellence in this regard, that the file can almost be entirely dispensed with as a separating instrument, especially in the back teeth. The heavier foil or gold is softer, and of consequence more adhesive than the thinner; with it therefore a more perfect filling can be made than with the thin; beneath the mallet it is plastic, may be made to spread out in the cavity, and so fill it perfectly. Adhesive gold in some form is rapidly taking the place of non-adhesive foil with all the more advanced in our profession; nearly all of our dental colleges have adopted it, the few who have not done so entirely, have to such an extent, that it can hardly be said that any of our educated young men are learning to fill teeth with non-adhesive gold. Among all the rapid strides in the way of improvement in the last quarter of a century, this is the greatest, and will be the grandest in results. When I reflect that nearly all of the young men now being introduced into our profession through regular channels; all who are taught in our colleges; all those who are to take prominent position, not only as practitioners but as teachers, repudiate the use of non-adhesive gold entirely or nearly so. I know it does not take a prophet, or the son of a prophet, to tell its fate, that in another decade it will be numbered with things which were and are not. Adhesive gold belongs to, and keeps company with improvements; as the mallet, rubber dam, burring engine, etc., etc. And he who will keep time to the march of dental science, must take hold upon it and adopt it, or ere long he will lie behind the rear guard; will no longer belong to the noble army now besieging the ramparts of ignorance, and seeking to carry blessings to suffering humanity; but will be a mere hanger on, a camp-follower, fit only to prey upon the weak, the ignorant, and defenseless.

